# Preparation and Application of Edible Chitosan Coating Incorporating Natamycin

**DOI:** 10.3390/polym17081062

**Published:** 2025-04-15

**Authors:** Jianming Sun, Tiantian Wang, Lei Liu, Qian Li, Hui Liu, Xiaofang Wang, Mengrui Liu, Han Zhang

**Affiliations:** 1 Department of Packaging Engineering, Henan University of Science and Technology, Luoyang 471023, China; sunjianming@haust.edu.cn (J.S.); wtt011518@163.com (T.W.);; 2 Henan Engineering Research Center of Intelligent and Protective Packaging Design, Luoyang 471023, China; 3 Henan Inspection and Testing Institute Group Co., Ltd., Zhengzhou 450018, China

**Keywords:** edible, chitosan, natamycin, coating, cherry tomatoes

## Abstract

In this paper, edible composite coatings, which used chitosan (CTS) as the matrix material, glycerol as the plasticizer, and natamycin as the antibacterial material, were prepared and composite films were prepared by a casting method. Taking cherry tomatoes as the research models, the optimal preservation effect of the composite coating was achieved using 10 g/L CTS, 2.5 g/L glycerol, and 125 mg/L natamycin under conditions of 25 °C and 50% RH. The thickness, transparency, water vapor transmittance (WVT), tensile strength (TS), and elongation at break (EB) of composite film were measured and the results showed the film prepared using 10 g/L CTS, 2.5 g/L glycerol and 125 mg/L natamycin was the best. The direct application of the optimal coating to cherry tomatoes kept the cherry tomatoes valuable for 20 days. The weight loss rate and hardness loss rate were reduced by 22.13% and 12.55%, respectively. The total soluble solid (TSS) content and vitamin c (Vc) content were increased by 2.54% and 20.35%, respectively. The malondialdehyde (MDA) content and peroxidase (POD) activity were decreased by 19.38% and 28.03%, respectively. Based on the significant preservation effect of the composite coating, it is expected to be widely used in the preservation of fruits and vegetables with skin morphologies similar to cherry tomatoes.

## 1. Introduction

Plastic wrap, a widely used material, is the dominant retail option for fruits and vegetables. In 2022, the global total plastic production reached 4.003 million tons, of which about 40% of plastics were used for packaging-related applications [1]. The processing and treatment of these plastics put tremendous pressure on the environment [2,3,4]. In addition, some materials have been banned from use in the food industry in some countries due to safety issues. In that sense, the fresh-keeping packaging industry urgently needs to transform towards ecological packaging [5]. Edible coatings are environmentally friendly and sustainable alternatives to plastic packaging materials. Applied through soaking, coating, or spraying, they can form a semi-permeable composite film on the surface of fruits and vegetables, reducing the gas exchange between fruits and vegetables and the environment [6]. They have good barrier [7], antibacterial [8,9], and antioxidant [10,11,12] properties and are widely studied in the preservation of fruits and vegetables [13].

CTS is derived via varying degrees of the deacetylation of chitin, an eco-friendly linear polysaccharide that is notable for its natural, biocompatible, and biodegradable properties. Additionally, CTS exhibits antimicrobial activity, which can be attributed to the presence of amine groups in its monomer units [14]. However, the application of the CTS coating is limited by its poor mechanical properties and ductility; therefore, it must be supplemented by incorporating other functional materials for food and vegetable packaging applications. Glycerol, characterized by an abundance of hydroxyl groups, exhibits strong hydrophilicity. Dong et al. [15] utilized CTS as the primary film-forming material and incorporated glycerol to prepare composite films. Various analyses, including microstructure, X-ray diffraction, thermogravimetric analysis, and tensile tests, were conducted to investigate the role of glycerol. The findings indicate that glycerol can be integrated into the molecular chain of CTS, disrupting the original crystalline structure. Consequently, the composite film demonstrates enhanced toughness and thermal stability compared to the pure CTS film at temperatures up to 200 °C.

Natamycin is a polyene macrolide derived from Streptomyces natalensis, which poses no safety risks. Upon ingestion, the body is capable of eliminating the majority of natamycin without experiencing toxic effects. It is a non-toxic, broad-spectrum antibiotic that is widely utilized in the food, medicine, and feed industries. Recent studies have primarily focused on the application of natamycin in blackberries [16], sweet potatoes [17], strawberries [18], and fresh-cut apples [19]. These investigations clearly demonstrate the practical utility of natamycin in preserving and maintaining the quality and shelf life of fruits and vegetables during post-harvest storage.

The cherry tomato is a species belonging to the Solanaceae family. These fruits are characterized by their short, pink-red, oval shape. As one of the most significant crops within the Solanaceae family [20], cherry tomatoes are widely consumed for their cellulose, protein and other essential nutrients, and also enhance the body’s resistance and possess various physiological functions, providing benefits for beauty and skin care. They are one of the “four major fruits” promoted by the Food and Agriculture Organization of the United Nations and are deeply favored by consumers. However, under abusive storage conditions, cherry tomatoes are easily affected by oxygen, moisture, microorganisms, causing them to spoil [21], resulting in unnecessary waste in terms of resources.

CTS is naturally sourced and non-toxic. It also meets food-grade standards. The amino groups in CTS’s molecular structure can disrupt microbial cell membranes, endowing it with broad-spectrum antibacterial activity. Its synergistic effect with natamycin significantly inhibits the growth of fungi and bacteria on fruit surfaces. Moreover, CTS can form a uniform and dense film in acidic conditions (such as 1% acetic acid solution). This film effectively blocks oxygen and moisture, inhibiting fruit respiration and water loss. Its mechanical strength and flexibility meet the preservation requirements at both room and refrigerated temperatures. Related studies (e.g., [22,23]) also confirm that CTS coatings perform excellently in maintaining cherry tomatoes firmness and inhibiting weight loss, with preservation effects superior to some synthetic films.

**Figure 1:**
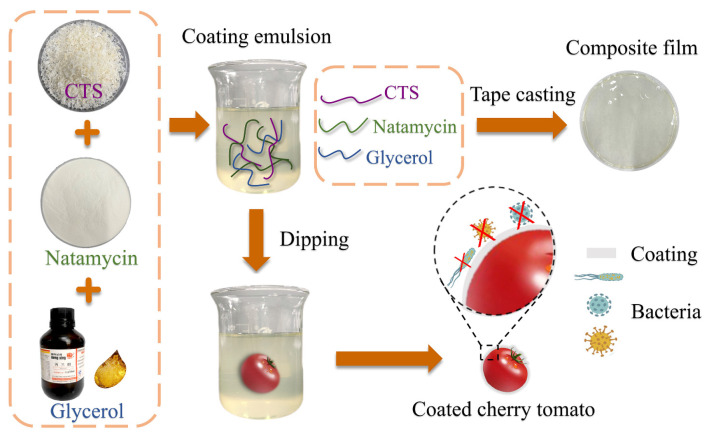
Experimental process and mechanism diagram.

Herein, we present a simple and effective strategy for the preparation of a biobased functional coating with excellent antibacterial properties in order to extend the shelf life of cherry tomatoes. As shown in Figure 1, CTS-based coatings were first prepared by mixing glycerol as the plasticizer and natamycin as the antimicrobial material to prepare an edible composite coating with certain mechanical and barrier properties. Then, the edible composite film prepared using CTS, glycerol, and natamycin was fully characterized (physicochemical, morphological, barrier properties) to assess its suitability for fruit and vegetable packaging applications. Given the versatility of CTS-based products, we speculated that coatings would yield versatile preservation coatings for cherry tomatoes in efforts to reduce the water loss, oxidation, and microbial invasion. To test our hypothesis, CTS-based coatings were applied on the surfaces of cherry tomatoes by a dip-coating procedure, and their preservation performance on the coated fruit was evaluated.

## 2. Materials and Methods

### 2.1. Materials

Cherry tomatoes were purchased from Mengjin County, Luoyang City, China. Samples were selected based on degree of ripeness, size and the absence of physiological damage in the form of defects. CTS (deacetylation degree > 90%, molecular weight 200 kDa) was purchased from Suzhou Tianke Trading Co., Ltd., Suzhou, China; natamycin (purity ≥ 98%) was purchased from Shanghai McLean Reagent Network, Shanghai, China. All other chemicals used in the study were purchased from Tianjin De’en Chemical Reagent Co., Ltd., Tianjin, China.

### 2.2. Preparation of Coatings

CTS, glycerol and natamycin were slowly dispersed in an acetic acid solution (1% *w*/*v*) in turn and we performed homogenization by means of mechanical stirring at 50 °C for 1.5 h or until complete dissolution. The specific coating solution composition is shown in Table 1.

### 2.3. Characterization of Coatings

#### 2.3.1. Preservation Effect

The coatings were applied to the surface of cherry tomatoes. The characterization of a coating’s preservation effect was carried out, with the weight loss rate and hardness loss rate of cherry tomatoes as the evaluation indicators. The weight loss rate was determined by weighing using an electronic balance (FA1004, Shanghai Sunny Hengping Scientific Instrument Co., Ltd., Shanghai, China). The hardness was measured using a digital fruit hardness tester (GY-4, Zhejiang Top Yunnong Technology Co., Ltd., Hangzhou, China).

#### 2.3.2. Coating Solution Viscosity

The coating solution’s viscosity was measured using a rotational viscometer (NDJ-1, Shanghai Pingxuan Technology Instrument Co., Ltd., Shanghai, China). The rotor was selected according to its range. After the rotor rotated several times in the solution, we pressed the pointer control lever. This was followed by us turning off the switch and reading the value when the pointer was in the reading window. Each type of procedure was repeated 10 times.

### 2.4. Preparation of Composite Film

We used a pipette to place 35 mL of coating solution on a horizontal circular panel (15 cm in diameter) to make it evenly cast. We put it in a desktop drying oven (202-0, Beijing Yongguangming Medical Instrument Co., Ltd., Beijing, China) and then peeled off the composite film after it was formed and stored it in a glass desiccator.

### 2.5. Characterization of Composite Film

#### 2.5.1. Thickness and Transparency

Composite films were first visually examined for their intactness. Using a digital thickness gauge (YHT112924, Shenzhen Yuanhengtongda Technology Co., Ltd., Shenzhen, China) with a sensitivity of 0.001 mm, at least 10 random spots on each film were measured and we averaged the results. The absorbance value at a wavelength of 600 nm was used to evaluate light transparency via triplicate samples of each type using a UV–visible spectrophotometer (UV759CRT, Shanghai Youke Instrument Co., Ltd., Shanghai, China). Rectangular composite film strips (10 mm × 40 mm) were stuck to glass cuvettes, where an empty one was used as a blank.

#### 2.5.2. Water Vapor Transmittance

The WVT of the films was determined by a desiccant method based on GB/T 1037-2021 [24] and ASTME96-2014 [25]. Round composite film samples (diameter of 8 cm) were mounted on top of plastic disks (diameter of 6 cm) containing 10 g of dried silica gel crystals. The samples were completely stuck to the disks with sealing wax (85% paraffin and 15% beeswax melted mixture). After we determined the initial weight, all the samples were placed in a chamber with a constant temperature and humidity (38 °C, 90% RH). The weight gain of disks was recorded periodically over time at a 24 h interval for 3 days. Three parallel samples were made for each film.

#### 2.5.3. Mechanical Properties

With reference to GB/T 1040.3-2006 [26], the tensile strength (TS) and elongation at break (EB) of composite film were measured by an intelligent electronic tensile testing machine (YT-L, Jinan Zhongce Electromechanical Equipment Co., Ltd., Jinan, China). Film samples were cut into rectangular-shaped (10 cm × 2 cm) strips clamped between two grips. The distance between two clamps was set to 60 mm according to the national standard, and the test was set at a speed of 50 mm/min. Six parallel samples were made for each group of composite film samples.

#### 2.5.4. Microstructure

The microstructure of the composite film was observed using an SEM (Smart Coater, Shenzhen Ruisheng Technology Co., Ltd., Shenzhen, China) operated at a set voltage. Film samples were cut into long strips, clamped with tweezers, placed in liquid nitrogen for 10 s to break them via external force, and coated with a thin gold layer for 2 min. When the vacuum value of the sample chamber reached 5 × 10^−4^ Pa, images of surface structure and cross-sections of films were obtained at different magnifications.

#### 2.5.5. Antibacterial Properties

(1) Zone of Inhibition

Using *Escherichia coli* (*E. coli*) as the indicator strain, the antibacterial activity of the composite film was evaluated by the agar diffusion method. The liquid and solid medium were prepared separately and then sterilized in an autoclave (121 °C, 20 min). Secondly, the frozen *E. coli* was inoculated into sterile liquid medium and incubated at 37 °C with shaking (180 rpm) for 18–24 h to reach the logarithmic growth phase. Then 1 mL of the said bacterial solution was taken, diluted with sterile saline to 0.5 McFarland standard (corresponding to a bacterial concentration of approximately 1.5 × 10^8^ CFU/mL), and then applied to the surface of the solid medium with the sterile L-shaped glass bar. The samples chosen for testing (polyethylene bag, optimized composite film) were placed onto the inoculated agar surface using sterile tweezers. The plates were inverted and incubated at 37 °C for 24 h. Inhibition zone diameters were measured using a vernier caliper, with triplicate measurements taken per sample.

(2) Dynamic changes in colony growth

The cherry tomatoes were washed with distilled water and dried; they were then divided into two groups. One group was wrapped with the optimized composite film as the experimental group, and the other was directly placed in a polyethylene bag as the control group. They were sealed and stored in a constant temperature and humidity chamber at 25 °C and 50% RH. The growth of the colonies was observed and recorded at regular intervals.

### 2.6. Cherry Tomatoes Preservation Experiment

#### 2.6.1. TSS Content

The peeled cherry tomatoes were ground into juice and then dripped into a handheld refractometer (LH-B55, Hangzhou Luheng Biotechnology Co., Ltd., Hangzhou, China) using a rubber-tipped dropper for measurement and data recording. The procedure was repeated three times and the results were averaged.

#### 2.6.2. Vc Content

The standard solution and reference solution were prepared according to the method of Khafid [27] to obtain the linear regression equation and draw the standard curve of ascorbic acid. Briefly, 10 g of peeled cherry tomato pulp was put into a crusher, and 10 mL of 5 g/L oxalic acid solution was poured and rapidly mixed into a homogenate. Then, we added distilled water; we shook and filtered the solution. Overall, 2 mL of 0.5 g/L oxalic acid solution was used as a reference. We measured filtrate absorbance at 243 nm and then calculated the Vc content in the sample.

#### 2.6.3. MDA Content

We followed the approach presented by Liu et al. [28], with some modifications. The peeled cherry tomato pulp was thoroughly ground in a mortar and then centrifuged in a high-speed refrigerated centrifuge (TGL-16, Sichuan Shuke Instrument Co., Ltd., Chengdu, China) until there was no turbidity. The supernatant was collected and used. The reaction system comprised 1.0 mL of enzyme solution and 1.0 mL of TBA (0.67%) solution. We used 1.0 mL of TCA (100 g/L) and 1.0 mL of TBA (0.67%) solution as the controls. After placing the mixed solution in a 100 °C water bath for 10–15 min, it was quickly cooled and transferred to a glass cuvette. The absorbance values were measured at 450 nm, 532 nm, and 600 nm, respectively.

#### 2.6.4. POD Activity

We worked according to the method of LIU [29], with certain modifications. The process involved peeling cherry tomato samples (4 g) with extraction buffe (4 mL) into a mortar. The pulp was ground in an ice bath and centrifuged until there was no turbidity. The resulting supernatant was collected for the subsequent examination. A test glass cuvette was prepared by adding 2.0 mL of guaiacol (25 mmol/L), 100 μL of enzyme solution, and 100 μL of H_2_O_2_ (0.5 mol/L). Then, it was put into the UV–visible spectrophotometer and the timer was started. Distilled water was used as the control group. The absorbance at 470 nm was determined, and subsequent absorbance readings were recorded every 30 s. An enzyme activity unit (U) was characterized by the change in the absorbance at 470 nm per unit time.

### 2.7. Statistical Analysis

Statistical analysis was performed using the SPSS statistics program (Version 22.0, Chicago, IL, USA) and graphs were constructed using Origin 9.1. Measurements denoted by the same lowercase letters indicate no significant difference (*p* > 0.05), while those with different lowercase letters indicate significant differences (*p* < 0.05). All experiments were carried out at least three times.

## 3. Results and Discussion

### 3.1. Characterization of Coatings

#### 3.1.1. Preservation Effect

**Figure 2:**
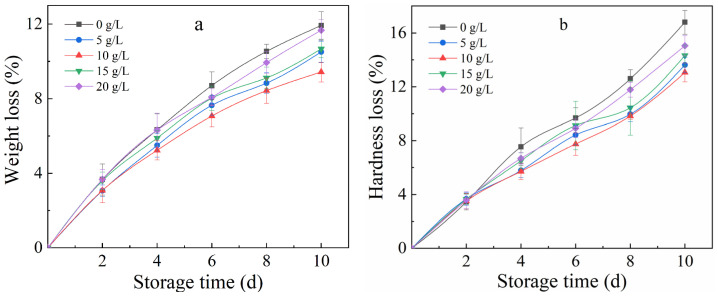

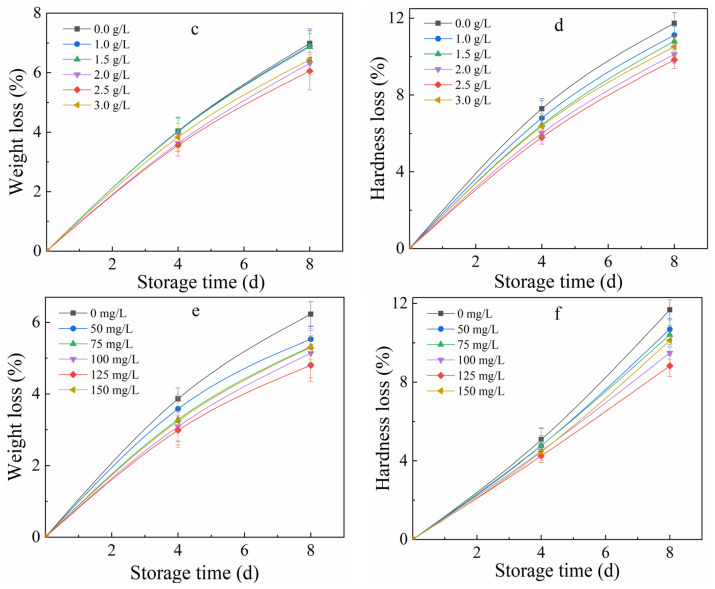
Coating preservation effect of CTS concentration (**a**,**b**), glycerol concentration (**c**,**d**), and natamycin concentration (**e**,**f**).

Cherry tomatoes lose weight, mainly due to the loss of water and carbon storage through transpiration [30] and respiration [31]. The weight loss (Figure 2a) rate continued to increase with the delay of time. Compared with the control group, the different concentrations of CTS groups all inhibited the weight loss of cherry tomatoes. After 4 days, the effect of 10 g/L CTS was the most ideal, followed by that of the 5 g/L CTS group, and the weight loss rates were reduced by 17.30% and 13.21%, respectively, compared with the control group; after 10 days of storage, the weight loss rate of 10 g/L CTS group decreased by 20.94%, which was significantly different from the results with control group (*p* < 0.05). These results can be attributed to the fact that CTS had good biological activities, such as film-forming, water-retaining, adsorption, and antibacterial properties. The coating could form a protective film on the surface of cherry tomatoes, slowing down water evaporation. During the 10-day storage, the hardness of cherry tomatoes decreased with time, and the hardness loss rate increased (Figure 2b). When stored for 2 days, the cherry tomatoes were not yet fully mature; the difference in hardness loss rate among each group was not significant. The hardness loss rate changed greatly in the middle period of storage (4–8 days), and the hardness of the control group was always higher than that of the CTS group; when stored for 10 days, the hardness loss rate of the 10 g/L CTS group decreased by 22.21%.

As can be seen in Figure 2c,d, the weight loss rate of the glycerol groups was always lower than that of the control group. This is mainly because glycerol is a small molecule with a hydrophilic group, and both the glycerol and CTS contain hydroxyl groups. The two molecules combine to form interchain hydrogen bonds [32], which reduce the intermolecular force, increasing the plasticity and flexibility of the film. After 4 days, there was no significant difference in the weight loss rate among the glycerol groups with different concentrations. On the 10th day, the effect of 2.5 g/L glycerol group was the best; the weight loss rate decreased by 13.37%, showing a significant difference (*p* < 0.05). The effect of glycerol concentration on the hardness loss rate began to show significant differences on the 4th day (*p* < 0.05). When the glycerol concentration was lower than 2.5 g/L, the performance of the coating had not reached its peak. On the contrary, the excess glycerol made the coating loose and sticky, which made the barrier properties relatively poor, resulting in a decrease in the hardness of the cherry tomatoes. This change trend is in accordance with the results of Pavinatto et al. [33] on the effect of glycerol on the barrier properties of CTS.

The effects of natamycin concentrations on the weight loss rate and hardness loss rate of cherry tomatoes are shown in Figure 2e,f. Both increased over time, but the results of the natamycin groups were always lower than those of the control group during the same period. This can be attributed to the fact that natamycin degrades fungal ergosterol and other sterol groups [34], destroys cell membrane permeability, causes cell leakage (amino acids [35], electrolytes [36] et al.) and death, reduces the decay of cherry tomatoes caused by microbial activity, and inhibits the loss of weight and the hardness of cherry tomatoes. As can be seen from the figure, after 8 days, there were significant differences among the groups (*p* < 0.05), among which the 125 mg/L natamycin group had the best effect; the weight loss rate and hardness loss rate were reduced by 22.91% and 24.38%, respectively, compared with the control group.

In conclusion, using weight loss rate and hardness loss rate as evaluation indicators to characterize the preservation properties of coatings, 10 g/L of CTS, 2.5 g/L of glycerol, and 125 mg/L of natamycin are found to be the optimal parameters.

#### 3.1.2. Dynamic Viscosity of Coating

**Figure 3:**
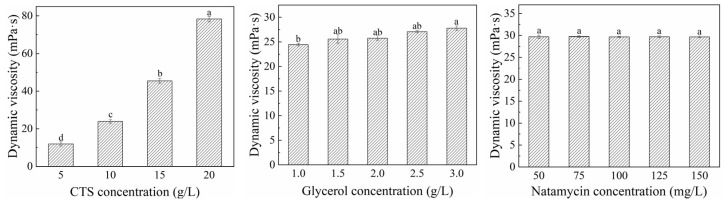
Effects of different concentrations of CTS, glycerol, and natamycin on dynamic viscosity of film fluid. Measurements denoted by the same lowercase letters indicate no significant difference (*p* > 0.05), while those with different lowercase letters indicate significant differences (*p* < 0.05).

Dynamic viscosity is able to reflect a coating’s fluidity and adhesion to a certain extent [37]. As shown in Figure 3, the viscosity of the coating solution gradually increased with higher amounts of CTS and glycerol, which might be attributed to the increased solute concentration creating greater resistance during rotor rotation, thereby significantly enhancing the dynamic viscosity. However, no significant differences were observed in viscosity among coating solutions with varying natamycin concentrations. This is likely because natamycin was added in minimal quantities (measured in mg), rendering its impact on viscosity negligible.

**Figure 4:**
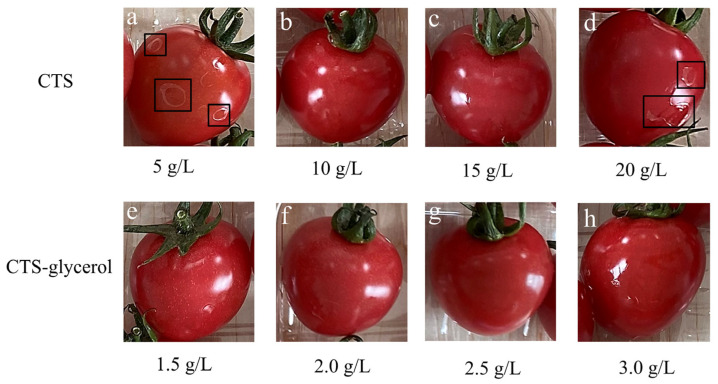
Photos of cherry tomatoes coated with different CTS concentrations (**a**–**d**) and glycerol concentrations (**e**–**h**). The black frame represent coating on the surface formed fragmented and patchy distributions.

Figure 4 shows photos of cherry tomatoes coated with different CTS, glycerol, and natamycin concentrations. It was found that coating solutions with excessively low CTS-based viscosity tended to form fragmented, patchy distributions on the fruit’s surface after drying, compromising film integrity and adhesion (Figure 4a). Conversely, solutions with overly high viscosity readily aggregated into blocks upon drying, impairing film uniformity and causing wrinkling or peeling when applied to produce surfaces (Figure 4d). Therefore, coating solutions with appropriate viscosity demonstrated optimal dispersion and film-forming capability, ultimately forming complete and well-adhered preservation coatings.

### 3.2. Characterization of Composite Film

#### 3.2.1. Thickness and Transparency

**Figure 5:**
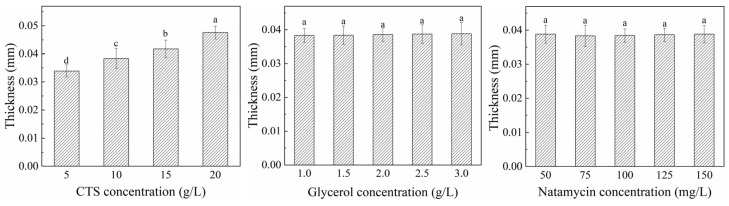
Effects of different concentrations of CTS, glycerol, and natamycin on thickness of composite film. Measurements denoted by the same lowercase letters indicate no significant difference (*p* > 0.05), while those with different lowercase letters indicate significant differences (*p* < 0.05).

**Figure 6:**
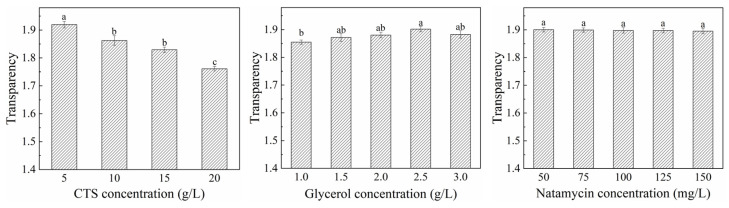
Effects of different concentrations of CTS, glycerol, and natamycin on transparency of composite film. Measurements denoted by the same lowercase letters indicate no significant difference (*p* > 0.05), while those with different lowercase letters indicate significant differences (*p* < 0.05).

The effects of CTS concentration, glycerol concentration, and natamycin concentration on the thickness and transparency of CTS-based composite film are illustrated in Figure 5 and Figure 6, respectively. As the CTS concentration increased, the thickness of the composite film significantly increased, while the transparency notably decreased at CTS concentrations of 10 g/L and 20 g/L. This phenomenon might be attributed to the higher CTS content imparting a yellowish hue to the composite film, which intensified with concentration. Additionally, the crystallinity (crystalline regions) of CTS in the composite film increased with higher CTS concentrations [38]. Light transmission paths were altered at the interfaces between crystalline and amorphous regions, leading to reflection or scattering effects and consequently reduced transparency [39].

The addition of glycerol showed no significant impact on film thickness. However, transparency initially increased and then slightly decreased with higher glycerol concentrations. This trend could be explained by glycerol molecules dispersing within the CTS molecular chains, reducing crystallinity and enhancing light transmittance [40]. Transparency reached its maximum value at approximately 2.5 g/L glycerol. Beyond this critical concentration, transparency slightly declined, likely due to partial glycerol phase separation, which increased light reflection [41].

The thickness of CTS-based composite film exhibited a slight but insignificant increase with higher natamycin concentrations, possibly due to tighter molecular packing induced by natamycin incorporation. As shown in Figure 6, transparency decreased marginally with increasing natamycin content, likely owing to the inherent cream-yellow color of natamycin, which deepened with concentration, thereby reducing light transmission [42]. Since the overall natamycin concentrations were low, their effects on both film thickness and transparency remained minimal.

#### 3.2.2. Water Vapor Transmittance

**Figure 7:**
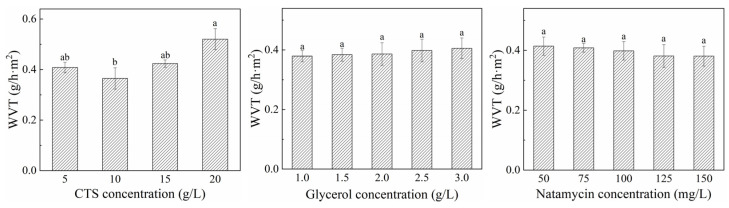
Effects of different concentrations of CTS, glycerol, and natamycin on water vapor transmittance of composite film. Measurements denoted by the same lowercase letters indicate no significant difference (*p* > 0.05), while those with different lowercase letters indicate significant differences (*p* < 0.05).

WVT is not only affected by the thickness of the film and the proportion of hydrophilic and hydrophobic groups, but is also closely related to the number of hydroxyl groups in the molecular chain [43] and the compatibility of the film components [44]. Within a certain range, the lower WVT of the film is beneficial to preservation [45]. The effects of varying concentrations of CTS, glycerol, and natamycin on the WVT of composite film are shown in Figure 7. WVT initially decreased and then increased with higher CTS concentrations. This trend might be attributed to the increased viscosity and thickness of the film-forming solution at elevated CTS levels, resulting in a denser film structure that impeded water vapor transmittance [46]. The minimum WVT value was observed at a CTS concentration of approximately 10 g/L. Beyond this point, the continued increase in CTS concentration enhanced interactions between positively charged amino groups and hydrogen bonds [47]. Furthermore, higher CTS concentrations increased hydroxyl group content, enhancing the hydrophilicity of the composite film and consequently raising WVT. A similar result was reported by Salama and Abdel Aziz [48] and Moreno et al. [49].

The addition of glycerol was found to improve WVT, with values increasing progressively as the glycerol concentration rose. This improvement might stem from glycerol breaking hydrogen bonds between CTS molecules, enhancing water permeability [50]. Additionally, glycerol acts as a plasticizer, loosening the composite film structure, thereby increasing WVT values [51]. In contrast, the incorporation of natamycin caused a slight decrease in WVT, likely due to its role in densifying the composite film structure, which reduced the diffusion rate of water vapor through the film’s interstitial spaces [52].

#### 3.2.3. Mechanical Properties

**Figure 8:**
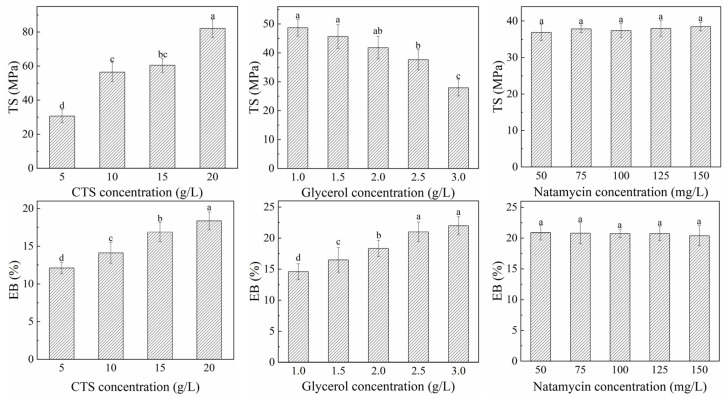
Effects of different concentrations of CTS, glycerol, and natamycin on mechanical properties of composite film. Measurements denoted by the same lowercase letters indicate no significant difference (*p* > 0.05), while those with different lowercase letters indicate significant differences (*p* < 0.05).

The mechanical properties of the films are reflected by TS and EB, which are affected by conditions and material processing methods [53,54,55]. The effects of varying concentrations of CTS, glycerol, and natamycin on the mechanical properties of composite film are shown in Figure 8. The TS and EB of the film increased with the increase in CTS. The mechanical properties seen using 10 g/L and 15 g/L of CTS concentrations were good, and there was no significant difference between them. With the addition of glycerol, the TS of the film decreased and the EB increased. Glycerol, as a small polyhydroxyl molecule, exhibits hydrophilic properties and reacts with the hydroxyl groups in the CTS molecules to produce interchain hydrogen bonds, softening the rigid structure of the CTS film [56]. This is manifested as an increase in the free volume between the macromolecules and their chain segments, thereby increasing the plasticity and elasticity of the film, causing the TS of the film to gradually decrease and the EB to increase. The concentration of natamycin had no significant effect on TS of the film and EB was only slightly reduced. This is mainly because the amount of natamycin added is very small and there is no specific structure that reacts with the CTS molecule.

#### 3.2.4. Microstructure

**Figure 9:**
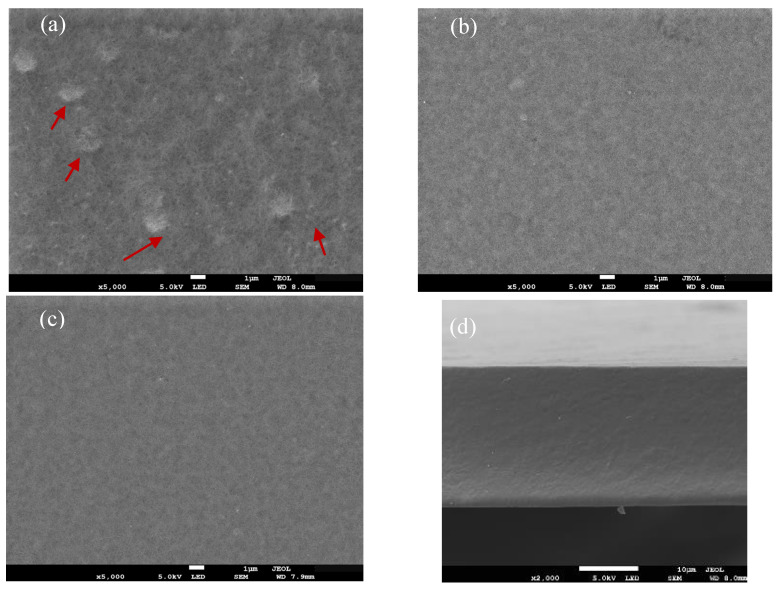
Scanning electron microscopy results of the CTS surface (**a**), the CTS–glycerol surface (**b**), and the optimized composite film surface (**c**), and a cross-sectional view of the optimized composite film (**d**).

Scanning electron microscopy (SEM) provides a direct and convenient approach to characterizing the microstructure and component compatibility of polymer composite film. As shown in Figure 9a–c, the surface morphologies of pure CTS film, CTS–glycerol composite film, and the optimized composite film are compared at 5000× magnification. The CTS film exhibited a smooth surface without visible bubbles, cracks, or pores, indicating excellent film-forming properties. However, as the CTS concentration increased, flaky protrusions emerged on the surface, accompanied by a significant rise in crystallinity [57]. Upon the addition of glycerol, the composite film surface became smoother and more homogeneous, with the disappearance of flaky structures, demonstrating strong compatibility between glycerol and CTS. This improvement can be attributed to glycerol molecules intercalating into the CTS molecular chains and forming new hydrogen bonds with amino and hydroxyl groups. These interactions disrupt the rigid crystalline structure of CTS, effectively suppressing the crystallization tendency and the brittle fracture of CTS films while improving interfacial stability [58]. Moreover, the low natamycin concentration in this study did not significantly alter the surface morphology of the composite film. Cross-sectional analysis of the optimized composite film (Figure 9d) revealed a uniform, dense, and pore-free layered structure, confirming the good compatibility among CTS, glycerol, and natamycin, as well as the formation of a stable multiphase system within the film.

#### 3.2.5. Antibacterial Properties

**Figure 10:**
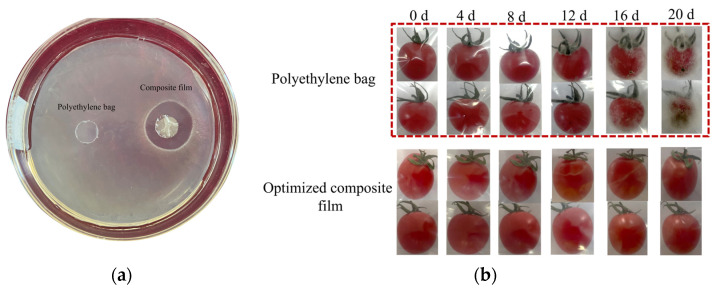
The antibacterial zone of the composite film (**a**) and the colonies growth of cherry tomatoes after 20 days of storage (**b**).

After harvest, fruits and vegetables are easily invaded by microorganisms such as bacteria and fungi and become spoiled. The antibacterial properties of edible coating materials can reduce the growth of microorganisms on the surface [59], slowing down physiological metabolism. Figure 10a demonstrates the antibacterial efficacy of the optimized composite film against *E. coli*. A distinct inhibition zone with an average diameter of 23.5 ± 0.15 mm was observed around the composite film, whereas no inhibitory effect was detected in the polyethylene bag. This antibacterial activity was attributed to CTS, the primary matrix of the composite film. Under acidic conditions, the amino groups (-NH_2_) of CTS undergo protonation to form positively charged moieties (-NH_3_^+^), which electrostatically interact with negatively charged components (e.g., lipopolysaccharides and teichoic acids) on bacterial cell walls. These interactions compromise membrane integrity, leading to the leakage of intracellular electrolytes and proteins, ultimately resulting in bacterial cell death [60,61,62]. Additionally, the synergistic effect between natamycin and CTS in the composite film may further enhance antimicrobial performance.

The optimized composite film wrapped group was used as the experimental group, and the polyethylene bag wrapped was used as the control group. The changes in cherry tomatoes over storage time are shown in Figure 10b. Colonies began to appear on the surface in the control group on the 10th day of storage and covered the entire surface as time went on. On the contrary, the experimental group had good gloss throughout storage, and no bacterial growth was found. This can be attributed to the composite film containing the antibacterial ingredient natamycin. Through hydrophobic action and hydrogen bonding, natamycin molecules are embedded into cell membranes and form transmembrane channels, resulting in damage to the integrity of cell membranes, the leakage of intracellular potassium ions, ATP and other small molecules, and ultimately cell death. Some studies have shown that natamycin can indirectly inhibit enzymes related to ergosterol synthesis and further damage the membrane structure [18,52,63]. Their combination can simultaneously inhibit bacteria and fungi, broadening the antimicrobial spectrum. Therefore, optimized composite film can effectively inhibit the growth of microorganisms during the storage.

### 3.3. Cherry Tomato Preservation Results

#### 3.3.1. Weight Loss Rate

**Figure 11:**
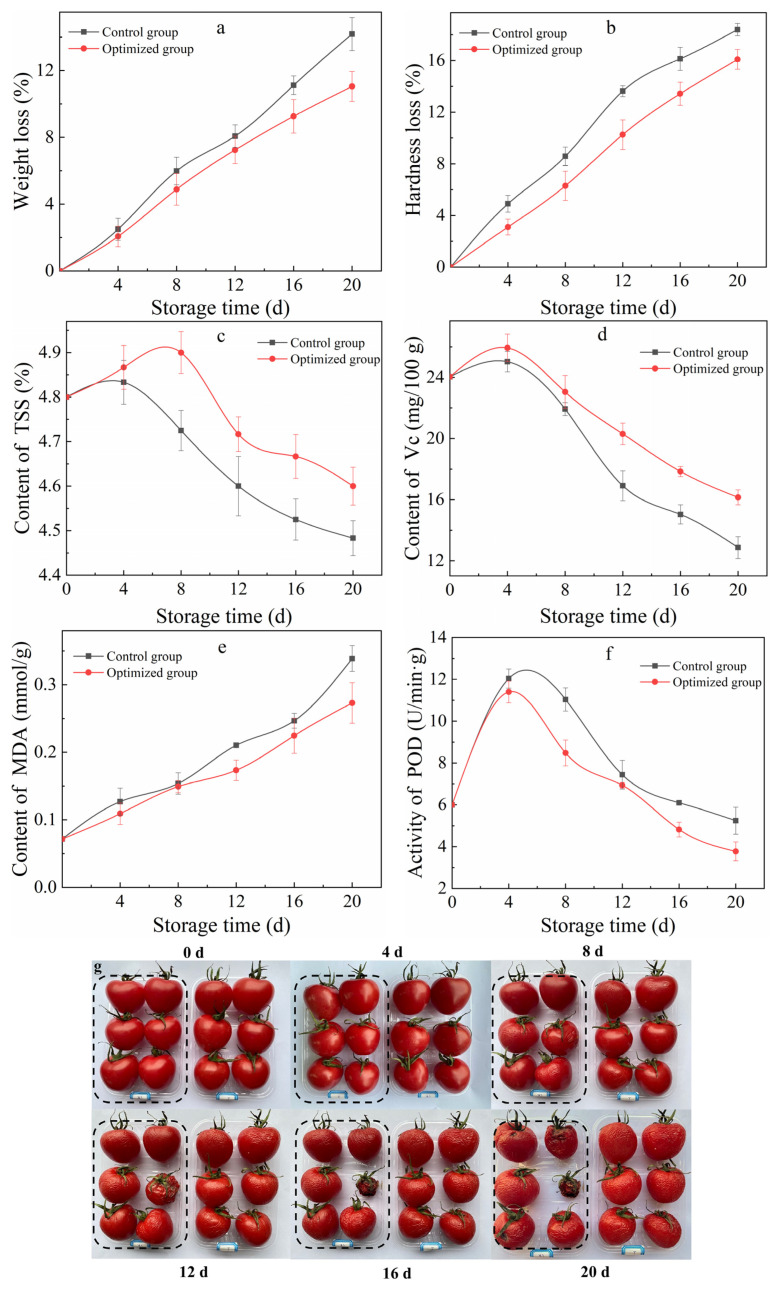
Effects of the optimized group on the weight loss rate (**a**), the hardness loss rate (**b**), TSS content (**c**), Vc content (**d**), MDA content (**e**), the POD activity of cherry tomatoes (**f**), and the preservation effect of cherry tomatoes after 20 days of storage (**g**).

The weight loss rate (Figure 11a) was positively correlated with storage time, and the increasing trend of the control group was faster than that of the optimized group. The coating reduces the internal loss of cherry tomatoes, and natamycin inhibits microbial activity [35], preventing the cherry tomatoes from rotting and deteriorating and thereby slowing down the aging process [64]. The data of the two groups began to show significant differences after 12 days (*p* < 0.05). On the 20th day, the weight loss rates of cherry tomatoes in the control group and the optimized group were 14.19% and 11.05%, respectively. The weight loss rate of the optimized group was 22.13% lower than that of the control group.

#### 3.3.2. Hardness Loss Rate

The hardness loss rate increased with storage time because cherry tomatoes undergo two changes during the ripening process. One change is a decrease in water content and peel surface shrinkage. The other is the cell walls that originally support the cells slowly degrade during the ripening of the fruit, which causes the pulp tissue structure to soften and loosen, resulting in decreasing hardness. As shown in Figure 11b, the hardness loss rate of the control group was always higher than that of the optimized group. After coating, a selectively permeable film was formed on the surface of the fruit, which created a low-O_2_ and high-CO_2_ environment [65] and delayed the softening and decay of the fruit. After 4 days, the hardness loss rate of the optimized group was 36.86% lower than that of the control group. After 20 days of storage, the data of the two groups were significantly different (*p* < 0.05).

#### 3.3.3. TSS Content

The main components of TSS are vitamins, organic acids, soluble sugars, and minerals [66], which directly affect nutritional quality. During the 20-day storage period, the TSS content of cherry tomatoes in each group first increased slowly and then decreased. At the beginning, the TSS content increased due to the increase in respiratory intensity and then tended to decrease due to the influence of respiration. The starch, pectin, and cellulose in the fruit were decomposed by enzymes and converted into soluble carbohydrates, causing the TSS content to continue to decrease [67]. As shown in Figure 11c, the TSS content of the optimized group was always significantly higher than that of the control group (*p* < 0.05), and the peak appeared later than that of the control group. Compared with the control group, the TSS content of the optimized group increased by 3.04% and 2.54% in the late stage of cherry tomatoes maturity (16 days and 20 days), respectively.

#### 3.3.4. Vc Content

Vitamin C, as an antioxidant and nutrient in cherry tomatoes, has a function of scavenging oxygen free radicals [68]. In Figure 11d, the Vc content shows a trend of first slowly increasing and then decreasing with time. Cherry tomatoes were not fully mature at the beginning, and the Vc content increased with the large amount of endogenous ethylene produced, reaching a peak after the cherry tomatoes matured. Later, the Vc was oxidized and lost activity; then, the content decreased. The Vc content of the optimized group was always higher than that of the control group, and the downward trend was relatively slight. The reason for this was that the coating reduced the gas exchange on the surface of the fruit, playing an important role in atmosphere preservation [69]. The addition of CTS and natamycin increased the activity of antioxidant enzymes, removing oxidative groups in fruit cells and reducing the oxidative decomposition of Vc. This change is in accordance with the results of Gangfeng et al. [70]. After 8 days of storage, the Vc content in the optimized group increased by 4.86%, and the statistical data showed a significant difference (*p* < 0.05).

#### 3.3.5. MDA Content

When cherry tomatoes are subject to stress, they produce a large amount of hydroxyl radicals and superoxide anion radicals, triggering cells lipid peroxidation [71], destroying cells components. MDA, as the main product [72], can measure the degree of peroxidation damage in cherry tomatoes cells [73]. As shown in Figure 11e, the MDA content of cherry tomatoes in each experimental group continued to increase with the increase in storage days, and the MDA content of the control group was always higher than that of the optimized group. After 4 days, the MDA content in the optimized group was 15.38% lower than that in the control group, showing a significant difference (*p* < 0.05).

#### 3.3.6. POD Activity

POD is an oxidoreductase that catalyzes the decomposition of H_2_O_2_ into O_2_ and H_2_O [74]. It also has the function of catalyzing or eliminating oxidative products such as phenols and aldehydes. Its activity reflects the ability of cells to scavenge free radicals [75]. In Figure 11f, the POD activity increased slowly and then gradually decreased during the entire storage period. The activity of the optimized group was always lower than that of the control group. This can be attributed to CTS and natamycin having a synergistic effect. By reducing the number of microorganisms on the surface of cherry tomatoes, the composite coating of CTS and natamycin can decrease the occurrence of diseases and infections [18,62], which in turn reduces the stress on the fruits and maintains the stability of POD activity. After 4 days, the POD activity in the optimized group was significantly lower than that in the control group (*p* < 0.05). Compared with the control group, the POD activity of the optimized group decreased by 21.00% and 28.03% in the late stage (16 days and 20 days), respectively.

#### 3.3.7. Preservation Effect

The preservation effect of cherry tomatoes after 20 days of storage is shown in Figure 11g. On the 8th day, the cherry tomatoes in the optimized group had good gloss, while the cherry tomatoes in the control group had sunken surfaces and gradually lost their commercial value. During the entire storage period, all cherry tomatoes experienced varying degrees of epidermal wrinkling, with the control group losing water significantly more. After 20 days of storage, the cherry tomatoes in the control group had severe water loss and shrinkage, with a large area of rot on the bottom. On the contrary, the optimized group only had slight wrinkles, with no microbial reproduction and rot.

## 4. Conclusions

This study systematically explored the effects of different concentrations of CTS, glycerol, and natamycin on the properties of the composite coating and films. It was found that when the CTS concentration was 10 g/L, the glycerol concentration was 2.5 g/L, and the natamycin concentration was 125 mg/L, the dynamic viscosity of the composite film solution, the film thickness, transparency, WVT, TS, and EB were 29.69 mPa s, 0.039 mm, 1.897, 0.38 g/h·m^2^, 37.99 MPa, and 20.75%, respectively. The composite film had moderate barrier properties, good mechanical strength, and a significant antibacterial effect.

The optimal formulation was applied to the preservation of cherry tomatoes, which achieving remarkable results. Compared with the control group, the shelf life of cherry tomatoes was extended to 20 days, and the weight loss rate and hardness loss rate were reduced by 22.13% and 12.55%, respectively, effectively maintaining the appearance and texture of cherry tomatoes. Meanwhile, the TSS content and Vc content were increased by 2.54% and 20.35%, respectively, indicating that the coating helped to preserve the nutritional quality. In addition, MDA content and the activity of POD were decreased by 19.38% and 28.03%, respectively, suggesting that the composite coating could effectively slow down the oxidative senescence process.

The research results indicate that this CTS-based composite coating has great potential in the preservation of cherry tomatoes, and it is expected to be widely applied in the preservation of fruits and vegetables with a high-water content and tendency to spoilage (strawberries, blueberries, lettuce, etc.), dairy products (cheese, yogurt, etc.), and fresh-cut fruits and vegetables. The future research direction can be divided into nanocomposite technology (the use of CTS nanoparticles to enhance natamycin stability and controlled release), multi-component synergy (the study of interactions with other natural antimicrobials to further enhance the preservation effect), and smart coatings (the development pH/humidity-responsive coatings for targeted release). However, research has currently only been carried out for a single variety of fruits and vegetables under specific temperature and humidity conditions. The coating preservation is mainly based on laboratory research, problems such as coating uniformity and drying time need to be solved in industrial production, and coating equipment suitable for large-scale production (such as electrostatic spraying) needs to be developed. Future research can be further expanded to environments with different temperatures and humidities and more types of fruits and vegetables in order to deeply explore the preservation mechanism of the composite coating and optimize the coating formulation, meeting diversified needs. Moreover, under high humidity or elevated temperatures, CTS may absorb moisture and soften, while natamycin’s photosensitivity could cause structural degradation, reducing the film’s mechanical properties (e.g., tensile strength loss) and long-term preservation efficacy. Therefore, long-term stability studies can be carried out to evaluate the performance changes of the composite coating under actual storage and transportation conditions, providing a more solid theoretical and practical basis for commercial application.

## Figures and Tables

**Table 1:** Coating solution composition and preparation steps.

**Solute**	**Solvent**	**Experiment Group**
CTS, 1% acetic acid	Distilled water	CTS concentrations of 0 g/L, 5 g/L, 10 g/L, 15 g/L, and 20 g/L
Glycerol	Optimized CTS coating solution	Glycerol concentrations of 0.0 g/L, 1.0 g/L, 1.5 g/L, 2.0 g/L, 2.5 g/L, and 3.0 g/L
Natamycin	Optimized CTS–glycerol composite coating solution	Natamycin concentrations of 0 mg/L, 50 mg/L, 75 mg/L, 100 mg/L, 125 mg/L, and 150 mg/L

## Data Availability

The original contributions presented in this study are included in the article. Further inquiries can be directed to the corresponding author.

## Abbreviations

CTS	chitosan
WVT	water vapor transmittance
TS	tensile strength
EB	Elongation at break
TSS	total soluble solid
Vc	vitamin C
MDA	malondialdehyde
POD	peroxidase
